# National Stroke Awareness Month — May 2014

**Published:** 2014-05-30

May is National Stroke Awareness Month. Stroke is the fourth leading cause of death in the United States (1). National Stroke Awareness Month aims to save lives by increasing awareness and educating the public about cardiovascular health. On average, one stroke-related death occurs every 4 minutes, or nearly 130,000 deaths each year (1). Approximately 800,000 persons a year will experience a stroke (2).

Anyone can have a stroke at any age. A person’s chances of having a stroke increase with certain risk factors, including high blood pressure, obesity, high cholesterol, a family history of stroke, age, and ethnicity. Risk for having a first stroke is nearly twice as high for blacks as for whites, and blacks are more likely to die after a stroke (2). Hispanics and American Indians/Alaska Natives also have a greater chance of having a stroke than do non-Hispanic whites or Asians (2).

During a stroke, every minute counts. Fast treatment can reduce the brain damage that stroke can cause. Signs of stroke include 1) sudden numbness or weakness in the face, arm, or leg, especially on one side of the body; 2) sudden confusion, trouble speaking, or difficulty understanding speech; 3) sudden trouble seeing in one or both eyes; 4) sudden trouble walking, dizziness, loss of balance, or lack of coordination; and 5) sudden severe headache with no known cause.

Persons should seek emergency care immediately if they or someone else has any of these symptoms (e.g., persons in the United States should immediately dial 9-1-1).

Stroke risk can be decreased by making healthy choices and managing health conditions. These behaviors include 1) eating a healthy diet; 2) maintaining a healthy weight; 3) getting enough physical activity; 4) not smoking; 5) limiting alcohol use; 6) getting blood pressure and cholesterol under control; and 7) taking medications as prescribed and working with a health-care team to prevent or treat the medical conditions that lead to stroke.

CDC’s Division for Heart Disease and Stroke Prevention focuses on promoting cardiovascular health, improving quality of care for all, and eliminating disparities associated with heart disease and stroke. More information is available at http://www.cdc.gov/bloodpressure and http://www.cdc.gov/stroke.
